# Incidence of Complications Following Coronary Intravascular Lithotripsy, Clinical Outcomes, and Predictors of Complications

**DOI:** 10.1016/j.jscai.2025.103706

**Published:** 2025-07-23

**Authors:** Martijn J.H. van Oort, Akshay A.S. Phagu, Federico Oliveri, Brian O. Bingen, Valeria Paradies, Gianluca Mincione, Bimmer E.P.M. Claessen, Aukelien C. Dimitriu-Leen, Tessel N. Vossenberg, Joelle Kefer, Hany Girgis, Frank van der Kley, J. Wouter Jukema, Ibtihal Al Amri, Jose M. Montero-Cabezas

**Affiliations:** a Department of Cardiology, Leiden University Medical Center, Leiden, the Netherlands; b Department of Cardiology, Maasstad Ziekenhuis, Rotterdam, the Netherlands; c Department of Cardiology, Amsterdam University Medical Center, Amsterdam, the Netherlands; d Department of Cardiology, Radboud University Medical Center, Nijmegen, the Netherlands; e Department of Cardiology, Frisius Medisch Centrum, Leeuwarden, the Netherlands; f Department of Cardiology, Cliniques Universitaires Saint-Luc, Brussels, Belgium; g Department of Cardiology, Jeroen Bosch Ziekenhuis, ‘s-Hertogenbosch, the Netherlands; h Netherlands Heart Institute, Utrecht, the Netherlands

**Keywords:** complication, coronary artery calcification, intravascular lithotripsy

## Abstract

**Background:**

This study aimed to evaluate the incidence of complications associated with intravascular lithotripsy (IVL) therapy, assess clinical outcomes, and identify predictors of complications in a real-world patient cohort.

**Methods:**

Patients undergoing IVL between May 2019 and September 2024 were enrolled from the BENELUX-IVL registry and categorized based on the occurrence of complications following IVL and concomitant therapy. End points were achievement of residual diameter stenosis <30% on quantitative coronary analysis and major adverse cardiovascular events (MACE) at 1-year follow-up. Kaplan-Meier and binary logistic regression analyses were performed to compare outcomes and to identify predictors of complications.

**Results:**

The study included 509 patients (73 ± 9 years, 75% male). Complications occurred in 33 patients (6%), of which 6 complications occurred immediately after IVL (1%). The most frequent complications were flow-limiting coronary dissections (n = 9, 2%), hemodynamic instability (n = 9, 2%), and coronary perforations (n = 7, 1%). These were effectively managed with 30 interventions, resulting in favorable procedural outcomes. Among patients with complications and available quantitative coronary analysis data, residual diameter stenosis <30% was achieved in 28 of 29 lesions (97%). One-year cumulative MACE was 11% (39 patients) and was significantly higher in patients with complications (*P* < .001), mainly driven by in-hospital events (*P* < .001). Larger predilatation balloon diameters (*P* = .032) were associated with complications.

**Conclusions:**

In this real-world registry, complications following IVL and concomitant therapy were infrequent. Patients with procedural complications had significantly higher 1-year MACE, primarily driven by in-hospital events. Larger predilatation balloon diameters were a significant predictor of complications.

## Introduction

The prevalence of coronary artery calcification (CAC) is increasing in patients undergoing percutaneous coronary intervention (PCI), currently observed in up to one-fourth of the cases.^1^^,^^2^ CAC may complicate PCI by increasing the risk of worse procedural outcomes, higher in-hospital major adverse cardiovascular event (MACE) rates, and worse long-term outcomes (in-stent restenosis, stent thrombosis, target vessel revascularization [TVR] and death)^1^^,^^3^, ^4^, ^5^, ^6^ Various plaque modification techniques have been developed to improve stent expansion and patient outcomes in the treatment of CAC.^7^, ^8^, ^9^

Intravascular lithotripsy (IVL) has emerged as a promising treatment for CAC, demonstrating safety and efficacy in previous studies.^10^ IVL utilizes a balloon-based system that generates shockwaves upon inflation at low pressure, targeting calcium deposits superficially and deeply embedded in the vessel wall. This facilitates calcium modification in the form of calcium fractures, which enables the use and expansion of balloons and stents in the affected calcified lesions.^10^, ^11^, ^12^ In the Disrupt CAD studies, the reported complication rates after IVL were low.^10^ However, these studies excluded patients presenting with acute coronary syndromes (ACS) and in higher-risk target lesion subsets, limiting generalizability to real-world populations.^13^ Thus, the incidence of complications related to IVL and concomitant therapy in an unselected patient cohort remains unclear. Moreover, it is unknown which factors may contribute to the occurrence of complications, and what their impact is on longer-term clinical outcomes. This study aimed to evaluate the rate of complications associated with IVL in an unselected real-world patient cohort, assess associated outcomes, and identify predictors of complications.

## Methods

### Population and data collection

This retrospective analysis used data from the all-comers international multicenter BENELUX-IVL registry (NCT06577038), in which patients (≥18 years) who underwent PCI for CAC with IVL were enrolled across 8 centers in 2 European countries between May 2019 and September 2024. Patients were categorized by the occurrence of complications. IVL was performed in all cases with the Shockwave Intravascular Lithotripsy Coronary System (Shockwave Medical). Technical aspects of the IVL procedure (eg, maximal inflation pressure and the number of pulses) and further treatment strategy (utilization of predilatation and postdilatation, supplementary stent placement, and use of intracoronary imaging [ICI] for procedural optimization) were left to the operator’s discretion. Demographic, clinical, procedural, and follow-up data were collected from the hospital's electronic records. Angiographic and ICI data were analyzed in a centralized core laboratory. The study was exempted by the Medical Research Ethics Committee Leiden Den Haag Delft (reference number: N22.199/HL/hl), and the retrospective analysis of clinically collected data was approved by the local ethical committees at each participating center.

### Definitions and imaging analysis

Coronary angiograms were evaluated in a centralized core laboratory. Coronary artery disease complexity was graded according to the SYNTAX score algorithm if all 3 vessels were recorded.^14^ CAC presence and severity were determined by the operator during the procedure both angiographically (fluoroscopic visibility of radiopacities in the vessel wall at the site of stenosis and/or noncompliant balloon underexpansion) and by ICI when available. Angiographically, CAC was scored none or mild, moderate (when the radiopacities were only visible during the cardiac cycle before contrast injection), or severe (when the radiopacities were apparent without cardiac motion before contrast injection).^8^^,^^15^ On intravascular ultrasound, CAC was defined as a hyperechoic signal with acoustic shadowing, whereas on optical coherence tomography CAC appeared as a signal-poor area with sharply delineated borders.^5^^,^^6^^,^^8^^,^^16^ On ICI, a calcium arc ≥270° was considered severe calcification.

Quantitative coronary analysis (QCA) and ICI were retrospectively analyzed offline to evaluate luminal gain following IVL. QCA was performed pre-IVL and post-IVL and stenting, using Medis Suite QCA (2D or 3D) software (Medis Suite 4.0.24.4; Medis Medical Imaging System BV). The decision to use ICI for treatment optimization was left to the operator. When available, analysis of intravascular ultrasound and optical coherence tomography was performed using QCU-CMS 4.69 (Leiden University Medical Center).

### Study end points

The primary end points were the occurrence of complications, including coronary dissections and perforations, hemodynamic instability requiring intervention, abrupt vessel closures, slow flow or no reflow, and resuscitation. Complications were assessed by the treating physician and documented in the patient records. To differentiate their origin, complications were categorized as either occurring immediately following IVL treatment or as related to the overall therapy, including concomitant therapies (eg, additional intervention strategies). This classification was further validated through a systematic retrospective analysis of the coronary angiograms by the centralized core lab. Secondary end points included the achievement of residual stenosis <30% on QCA and the occurrence of MACE (cardiac death, nonfatal target vessel myocardial infarction, or clinically driven TVR) up to 1-year follow-up.

### Statistical analysis

Continuous variables are presented as either the mean ± SD or median with IQR (25th to 75th percentile), as appropriate. Differences between unpaired continuous variables were assessed with the unpaired *t* test if normally distributed, and with the Mann-Whitney *U* test if not normally distributed. Categorical variables were reported as frequencies and percentages and analyzed using the χ^2^ test for expected counts ≥5 per cell, whereas Fisher exact test was applied for variables with expected counts <5 per cell. Kaplan-Meier analysis was performed to estimate cumulative survival free of MACE at 1-year follow-up and the results were compared between groups using the log-rank test. Based on clinical relevance, the relationships between selected clinical and procedural variables and the occurrence of complications were analyzed using univariable binary logistic regression. Variables with a *P* value <0.1 on univariate analysis were entered in multivariable models to account for potential confounding factors and identify factors independently associated with complications. Results from the binary logistic regression are reported as odds ratios (OR) with 95% CI and *P* values. Statistical significance was defined as a 2-sided *P* value <.05. All statistical analyses were performed with SPSS for Windows version 25.0 (IBM Corp).

## Results

### Complications and management

The patient cohort consisted of 509 patients (mean age 73 ± 9 years, 75% male) with a total of 537 calcified target lesions treated. Overall, complications occurred in 33 patients (6%), and 33 lesions occurred immediately after IVL (1%).

The most frequent complications were flow-limiting coronary dissections (n = 9, 2%) and hemodynamic instability (n = 9, 2%), followed by coronary perforations (n = 7, 1%) and abrupt vessel closures (n = 4, 1%) (Table 1 and Central Illustration).

**Table 1:** Complications and interventions.

	Overall (N = 509)
Complications	33 (6)
Coronary dissection	9 (2)
Coronary perforation	7 (1)
Slow flow	1 (0.2)
No reflow	2 (0.4)
Abrupt vessel closure	5 (1)
Hemodynamic instability	9 (2)
Reanimation	5 (1)
Other	5 (1)
Immediately after IVL	6 (1)
Intervention performed	30 (6)
Drug-eluting stent	9 (2)
Covered stent	6 (1)
Drug-eluting balloon	2 (0.4)
Inotropic support	9 (2)
Vasopressive support	6 (1)
Mechanical support	3 (1)
Reanimation	5 (1)
Pericardial puncture	1 (0.2)
Thoracic surgery	2 (0.4)
Defibrillation	1 (0.2)
Temporary pacemaker	1 (0.2)

Values are n (%). IVL, intravascular lithotripsy.

**Central Illustration:**
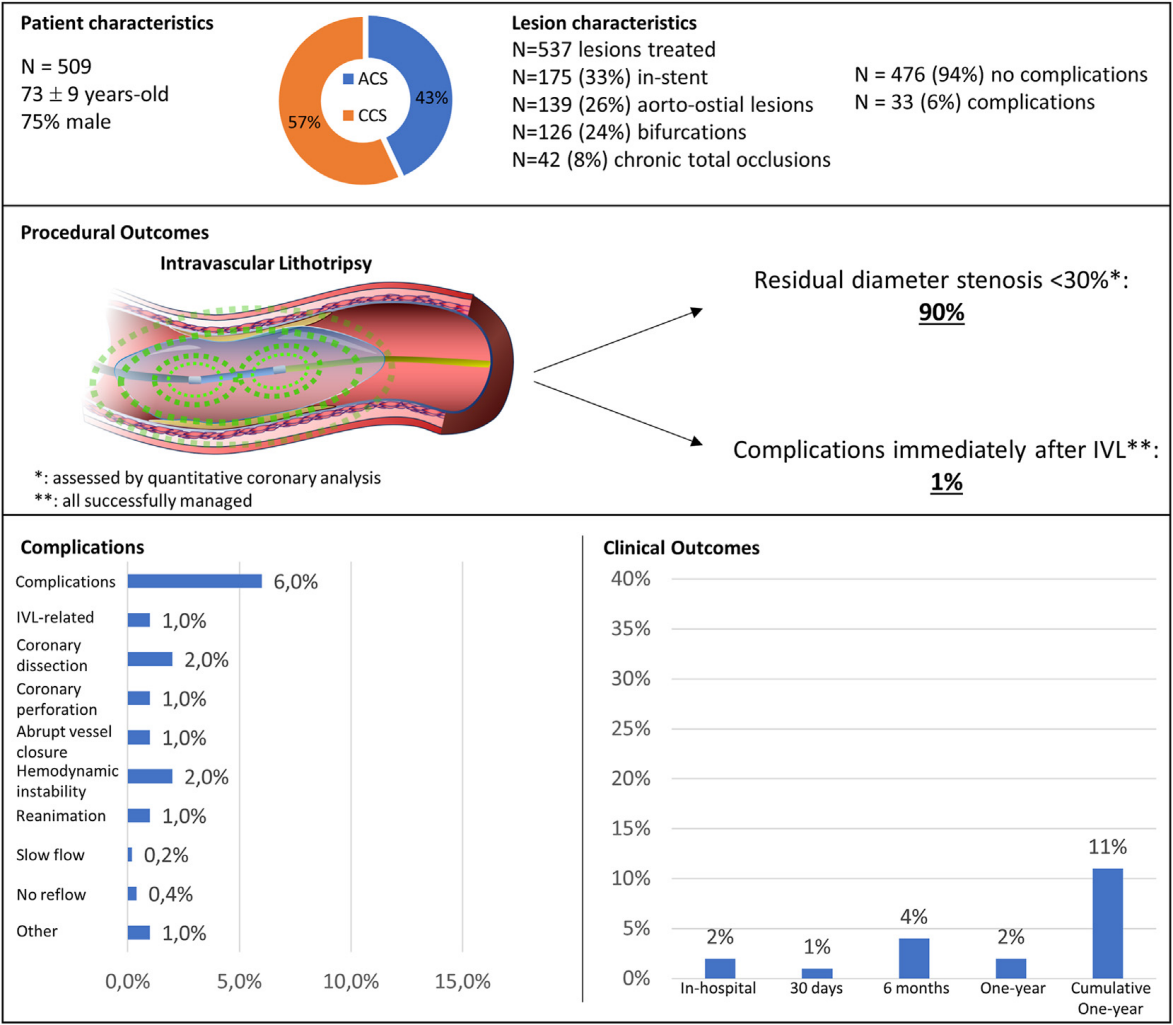
**Patient and lesion characteristics, procedural outcomes, complications, and clinical outcomes following coronary intravascular lithotripsy (IVL).** ACS, acute coronary syndrome.

Interventions were required in 30 patients (6%) (Table 1). Coronary dissections and abrupt vessel closures were predominantly treated with implantation of drug-eluting stents (n = 9). Hemodynamic instability (n = 9, 2%) was managed with inotropics (n = 9, 2%), vasopressors (n = 6, 1%), and in severe cases, mechanical circulatory support (n = 3, 1%) or resuscitation (n = 5, 1%). Coronary perforations were primarily treated with covered stents (n = 6), with 1 case requiring additional mechanical circulatory support, pericardial puncture, and cardiac resuscitation. Severe complications, such as an aortic root dissection (n = 1) and an unmanageable dissection of a large diagonal branch (n = 1), necessitated urgent thoracic surgery (n = 2, 0.4%). A case of slow flow in a side branch was treated with drug-coated balloon inflation. Rhythm complications were rare, including ventricular fibrillation (n = 1, <1%) requiring defibrillation, and total atrioventricular block (n = 1, <1%) requiring a temporary pacemaker. Additionally, 3 complications (1%) were managed conservatively: 1 abrupt vessel closure, a vascular access site dissection, and no-reflow phenomenon following rotational atherectomy (RA).

The complications occurring immediately after IVL included 2 controlled coronary dissections (0.4%) potentially caused by calcium fractures, 2 cases of hemodynamic instability (0.4%), 1 coronary perforation due to IVL balloon rupture (0.2%), 1 abrupt vessel closure (0.2%), 1 patient requiring cardiac resuscitation (0.2%), and 1 episode of peri-procedural ventricular fibrillation (0.2%). All these complications were successfully managed.

### Baseline characteristics

Baseline characteristics and medical history were comparable between the complication and noncomplication groups (Table 2). A trend toward a higher percentage of female patients with complications was observed (39% vs 24%; *P* = .051). Two hundred nineteen (43%) patients presented with ACS and 290 (57%) with chronic coronary syndrome, and were comparable between both groups (*P* = .424).^17^ The mean SYNTAX score was 21.7 ± 13.3 and was comparable between groups (*P* = .831).

**Table 2:** Baseline demographics and medical history.

	Overall (N = 509)	No complication (n = 476)	Complication (n = 33)	*P* value
Age, y	73.0 ± 9.1	72.9 ± 9.2	74.1 ± 8.2	.451
Male sex	381 (75)	361 (76)	20 (61)	.051
Diabetes mellitus	169 (33)	159 (33)	10 (30)	.759
Hypertension	358 (70)	333 (70)	25 (76)	.427
Hypercholesterolemia	279 (55)	263 (55)	16 (49)	.573
Family history of CAD	124 (24)	115 (24)	9 (27)	.770
Chronic kidney disease^a^	157 (31)	147 (31)	10 (30)	.844
SYNTAX score	21.7 ± 13.3	21.7 ± 13.4	22.3 ± 13.0	.831
Fluoroscopic calcification (n = 366)				.543
Not possible	48 (13)	47 (14)	1 (4)	
None/mild	21 (6)	19 (6)	2 (9)	
Moderate	35 (10)	32 (9)	3 (13)	
Severe	262 (72)	245 (71)	17 (74)	
Left ventricular ejection fraction, % (n = 398)	50 (38-59)	50 (38-59)	45 (20-53)	.230
Smoking history	210 (41)	198 (42)	12 (36)	.475
Previous stroke	74 (15)	71 (15)	3 (9)	.604^b^
Previous MI	196 (39)	186 (39)	10 (30)	.424
Previous PCI	245 (48)	230 (48)	15 (46)	.997
Previous CABG	98 (19)	92 (19)	6 (18)	.997
Clinical presentation				.424
ACS	219 (43)	207 (43)	12 (36)	
CCS	290 (57)	269 (57)	21 (64)	

Values are mean ± SD, n (%), or median (IQR). ACS, acute coronary syndrome; CABG, coronary artery bypass graft surgery; CAD, coronary artery disease; CCS, chronic coronary syndrome; MI, myocardial infarction; PCI, percutaneous coronary intervention.

a Estimated glomerular filtration rate <60 mL/min/1.73 m^2^.

b Fisher exact test.

### Procedural characteristics

The lesion and procedural characteristics are displayed in Table 3. IVL balloons had a median diameter of 3.5 (3.0-4.0) mm and delivered 80 (60-80) pulses per procedure. Predilatation before IVL (n = 495, 92%) and postdilatation after IVL (n = 500, 93%) were performed in most of the procedures. Additional plaque modification techniques alongside IVL were used in 85 (16%) lesions, of which 77 (14%) were performed before IVL and 9 (2%) after IVL. Pre-IVL plaque modification consisted of RA (n = 70), cutting (n = 5), scoring (n = 1), and ultrahigh pressure (OPN) balloon inflation (n = 1). Post-IVL, RA (n = 5), cutting (n = 1), and OPN balloons (n = 3) were used. ICI was used for procedural optimization in 284 lesions (53%). Procedural time (110.3 ± 52.6 min vs 93.4 ± 80.2 min; *P* = .244), fluoroscopy time (31.7 ± 18.1 min vs 28.6 ± 18.3 min; *P* = .380), and contrast volume (204.1 ± 105.5 mL vs 181.3 ± 72.6 mL; *P* = .115) were slightly higher in patients with complications; however, these differences did not reach statistical significance.

**Table 3:** Procedural and lesion characteristics.

	Overall (N = 537)	No complication (n = 504)	Complication (n = 33)	*P* value
Target vessel				
Left main coronary artery	62 (12)	61 (12)	1 (3)	.158^a^
Left anterior descending artery	240 (45)	227 (45)	13 (39)	.527
Left circumflex artery	85 (16)	79 (16)	6 (18)	.702
Right coronary artery	188 (35)	174 (35)	14 (42)	.357
Bypass graft	5 (1)	5 (1)	0	1.000^a^
Lesion characteristics				
Bifurcation	126 (24)	116 (23)	10 (30)	.339
Ostial	139 (26)	133 (26)	6 (18)	.297
Chronic total occlusion	42 (8)	38 (8)	4 (12)	.315^a^
Long-segment	346 (64)	322 (64)	24 (73)	.304
In-stent	175 (33)	166 (33)	9 (27)	.501
IVL				
Balloon diameter, mm	3.5 (3.0-4.0)	3.5 (3.0-4.0)	3.5 (3.0-4.0)	.577
No. of pulses	80 (60-80)	80 (60-80)	80 (50-80)	.503
Predilatation	495 (92)	467 (93)	28 (85)	.076^a^
Pressure, atm	19.4 ± 4.4	19.4 ± 4.3	18.4 ± 6.1	0.248
Balloon diameter, mm	3.0 (2.5-3.5)	3.0 (2.5-3.5)	3.5 (2.5-4.0)	.216
Postdilatation	500 (93)	470 (93)	30 (91)	.448^a^
Pressure, atm	19.5 ± 4.5	19.5 ± 4.4	19.1 ± 5.9	.705
Balloon diameter, mm	3.5 (3.5-4.0)	3.5 (3.5-4.0)	4.0 (3.5-4.25)	.564
After stenting (bail-out)	82 (15)	77 (15)	5 (15)	0.984
ICI performed	284 (53)	262 (52)	22 (67)	.107
Pre-IVL	218 (41)	201 (40)	17 (52)	.187
Post-IVL	238 (44)	225 (45)	13 (39)	.557
Other plaque modification technique used	85 (16)	79 (16)	6 (18)	.710
Pre-IVL	77 (14)	71 (14)	6 (18)	.452^a^
Rotational atherectomy	70 (13)	64 (13)	6 (18)	.419^a^
Cutting balloon	5 (1)	5 (1)	0	1.000^a^
Scoring balloon	1 (0.2)	1 (0.2)	0	1.000^a^
OPN balloon	1 (0.2)	1 (0.2)	0	1.000^a^
Post-IVL	9 (2)	9 (2)	0	1.000^a^
Rotational atherectomy	5 (1)	5 (1)	0	1.000^a^
Cutting balloon	1 (0.2)	1 (0.2)	0	1.000
OPN balloon	3 (0.6)	3 (0.6)	0	1.000
Treatment after IVL				
Stent implantation	412 (77)	389 (77)	23 (70)	.324
Drug-coated balloon	40 (7)	38 (8)	2 (6)	1.000^a^
Procedural time, min	94.6 ± 78.8	93.4 ± 80.2	110.3 ± 52.6	.244
Fluoroscopy time, min	28.8 ± 18.3	28.6 ± 18.3	31.7 ± 18.1	.380
Contrast volume, mL	182.8 ± 75.2	181.3 ± 72.6	204.1 ± 105.5	.115

Values are mean ± SD, n (%), or median (IQR). ICI, intracoronary imaging; IVL, intravascular lithotripsy; OPN, ultrahigh pressure balloon; RA, rotational atherectomy; RCA, right coronary artery.

a Fisher exact test.

### Outcomes

After therapy completion, residual diameter stenosis <30%, as assessed by QCA, was achieved in 394 of 437 lesions (90%) available for analysis. This included 28 of 29 lesions (97%) in patients with complications and 366 of 408 lesions (90%) in patients without complications, with no significant difference between groups (*P* = .341).

**Figure 1:**
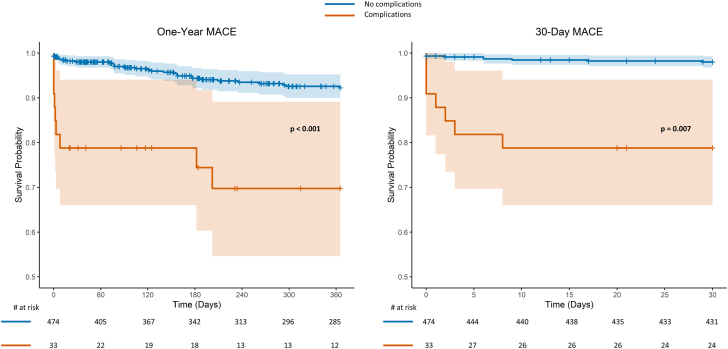
**Comparison of 30-day and 1-year major adverse cardiovascular events (MACE) between patients with and without complications**.

One-year follow-up was completed in 346 patients (68% of the total cohort), including 25 of 33 patients (76%) with complications and 325 of 476 patients (68%) without complications. MACE occurred in 39 (11%), with a significantly higher cumulative rate in patients with complications (n = 9, 43% vs n = 30, 9%; *P* < .001) at 1-year follow-up (Table 4). The 1-year survival probability was significantly lower in patients with complications (log-rank *P* < .001) (Figure 1). This difference was primarily driven by in-hospital MACE, which occurred in 10 patients overall (2%): 6 (18%) in patients with complications and 4 (1%) in patients without complications (*P* < .001). The in-hospital events included 8 cardiac deaths (5 in patients with complications and 3 in patients without complications) and 2 TVR (1 case in each group).

**Table 4:** Clinical outcomes.

	Follow-up complete (Overall, No-C, C)	Overall (N = 509)	No complication (n = 476)	Complication (n = 33)	*P* value
In-hospital MACE	509, 476, 33	10 (2)	4 (1)	6 (18)	<.001^a^
30-Day MACE	476, 444, 32	6 (1)	5 (1)	1 (3)	.343^a^
6-Month MACE	397, 371, 26	15 (4)	14 (4)	1 (4)	1.000^a^
1-Year MACE	346, 325, 21	8 (2)	7 (2)	1 (5)	.397^a^
Cumulative 1-year MACE	346, 325, 21	39 (11)	30 (9)	9 (43)	<.001

Values are n (%) occurring within each time period. The cumulative 1-year MACE includes all events from hospitalization up to 1 year.

C, complications; MACE, major adverse cardiovascular events; No-C, no complications.

a Fisher exact test.

### Risk factors for complications

The multivariate binary logistic analysis identified higher balloon-to-artery ratio (BAR)—defined as the ratio between the diameter of the pre-IVL predilatation balloon and the reference vessel diameter as measured by QCA—as significantly associated (OR, 1.453; 95% CI, 1.074-1.966; *P* = .015) with the occurrence of complications (Table 5). The overall BAR was 0.9 (0.8-1.0), with a nonsignificant trend toward higher values in patients with complications (1.0 [0.8-1.1] vs 0.9 [0.8-1.0]; *P* = .511). Additional plaque modification alongside IVL was not associated with the occurrence of complications (OR, 1.528; 95% CI, 0.607-3.844; *P* = .368).

**Table 5:** Univariate and multivariate binary logistic regression analysis.

Variable	Univariate analysis			Multivariable analysis		
	Odds ratio	95% CI	*P* value	Odds ratio	95% CI	*P* value
Age	1.016	0.976-1.057	.450			
Sex (male)	0.490	0.236-1.016	.055	0.563	0.231-1.376	.208
Body mass index	0.906	0.826-995	.039	0.917	0.829-1.015	.096
Aorta-ostial lesion	0.620	0.250-1.535	.301			
Chronic total occlusion	1.691	0.565-5.063	.347			
Bifurcation	1.454	0.673-3.144	.341			
Long-segment	1.507	0.686-3.312	.307			
In-stent lesion	0.764	0.347-1.680	.502			
Procedural characteristics						
Maximum IVL inflation pressure	0.924	0.650-1.312	.657			
No. of IVL pulses delivered	1.002	0.988-1.017	.743			
Maximum pressure	0.951	0.873-1.035	.246			
Maximum balloon size	1.095	1.005-1.193	.039			
Balloon-to-artery ratio	1.382	1.034-1.845	.029	1.453	1.074-1.966	.015
Post-IVL maximum pressure dilatation	0.983	0.900-1.074	.704			
Maximum balloon size	1.046	0.925-1.182	.473			
Plaque modification next to IVL	1.190	0.476-2.976	.710			
Stenting after IVL	0.680	0.315-1.470	.327			
Intracoronary imaging performed	1.832	0.870-3.858	.111	1.574	0.672-3.687	.296

Maximum predilatation balloon size was excluded from the multivariate analysis due to multicollinearity with the balloon-to-artery ratio.

IVL, intravascular lithotripsy.

## Discussion

This study aimed to evaluate the rate of complications associated with IVL using data from the BENELUX-IVL registry, assess clinical outcomes, and identify predictors of complications. The main findings include the following: (1) a low complication rate after IVL and subsequent therapy (6%); (2) good procedural outcomes, with 90% of patients achieving residual stenosis <30%; (3) a higher cumulative 1-year MACE rate in patients with complications compared to patients without complications, mainly driven by in-hospital events; and (4) higher BAR as a predictor of complications.

Complications after IVL and subsequent therapy were infrequent (6%), and were even lower when assessing complications occurring immediately after IVL (1%). Importantly, these complications were managed successfully during the procedure, resulting in good procedural outcomes reflected by residual diameter stenosis <30% on QCA achieved in 90% of the patients, and this was comparable for patients in the complication and noncomplication group. Moreover, these good procedural results were achieved in a complex patient cohort, as reflected by the high SYNTAX score, the patient presentations including ACS, and the variety of target lesion subsets treated. This reinforces the efficacy and safety of IVL in a real-world setting.

Despite the favorable procedural outcomes, the cumulative 1-year MACE rate was higher in patients who experienced complications, primarily driven by in-hospital events (Figure 1). Among 8 cardiac deaths, 5 occurred in patients with complications and 3 in patients without complications. These were caused by a cardiogenic or hypovolemic shock in 5 cases, sudden cardiac arrest in 2 cases, and an iatrogenic aortic root dissection after high-pressure postdilatation in 1 case. TVR was performed in 1 patient of each group; 1 required emergency coronary artery bypass graft surgery for coronary dissection after predilatation while the other underwent PCI for thrombus embolization. Although the comparison of clinical outcomes should be interpreted with caution due to the limited sample size of patients with complications and the low number of events, these findings underscore the need for further efforts to prevent complications. Notably, our analysis identified a higher BAR as being associated with the occurrence of complications. This finding suggests that more precise balloon sizing may help mitigate this risk. This may potentially be achieved through increased use of ICI to tailor lesion preparation strategies and ensure an appropriate BAR.

The 6% complication rate observed in this study is consistent with findings from other studies evaluating IVL. In the pooled analysis of the Disrupt CAD studies, a lower complication rate was reported at 2%.^10^ However, in this study patients with ACS and complex target lesion subsets were excluded, which may have contributed to the lower complication rate compared to our results. In contrast, the REPLICA-18 study reported an identical complication rate of 6% and included a significant proportion of patients presenting with ACS.^18^ This consistency suggests that IVL is applicable and effective in real-world patient populations.

When comparing IVL to calcium modification techniques, such as RA and orbital atherectomy (OA), procedural success rates appear comparable, whereas complication rates tend to be lower with IVL. ROTAXUS reported a 93% success rate for RA, while ORBIT II showed a 90% success rate for OA, similar to the 89% success rate reported for IVL.^19^, ^20^, ^21^ In ROTAXUS, the complications occurred at similar rates compared to this study (7% in ROTAXUS vs 6% in this study). In ORBIT II, dissections (6%) and perforations (2%) were more frequent compared to IVL (2% and 1%, respectively). However, cross-trial comparisons are limited due to differences in inclusion criteria and the time periods in which they were conducted. More recently, the ECLIPSE trial (comparing OA with balloon angioplasty) showed comparable procedural success rates to this study, with numerically higher rates of dissection (6.9%) and slow flow (1.4%), and comparable rate of perforations (1.8%).^22^ Considering IVL’s ease of use and shorter learning curve compared to RA and OA, it appears to be a safer alternative to these techniques if it can be delivered, but randomized trials are needed to demonstrate this conclusively. Several trials such as the ISAR-WAVE trial are ongoing that compare IVL to other plaque modification techniques which are expected to provide further insights into the optimal approach for calcified coronary lesions.^23^

### Limitations

This study has several limitations. First, the retrospective observational design limits the ability to evaluate the clinical outcomes and understand the mechanism of occurrence of complications. Second, the relatively low number of patients with complications limits the statistical power for direct group comparisons. This limits the robustness of subgroup analyses and may affect the generalizability of the findings. Lastly, ICI was not routinely performed before and after IVL, which limits the ability to evaluate the impact of ICI on procedural and clinical outcomes. Therefore, the outcomes of this study should be interpreted as hypothesis-generating, warranting further validation in prospective randomized trials.

## Conclusion

In this real-world registry, complications following IVL and concomitant therapy were infrequent. Patients who experienced complications had a significantly higher cumulative MACE rate at 1-year follow-up, primarily driven by in-hospital events. Higher BAR were significantly associated with the occurrence of complications. Future randomized studies are needed to validate these findings.
